# Attitudinal analysis of vaccination effects to lead endemic phases

**DOI:** 10.1038/s41598-023-37498-y

**Published:** 2023-06-24

**Authors:** Donggyun Ku, Gahyun Kim, Kyong Ran Peck, In Kwon Park, Rakwoo Chang, Donghan Kim, Seungjae Lee

**Affiliations:** 1 University of Seoul, Seoul, South Korea; 2 Division of Infectious Diseases, Samsung Medical Centre, Sungkyunkwan University School of Medicine, Seoul, South Korea; 3 Seoul National University, Seoul, South Korea; 4 Korea Research Institute for Human Settlements, Sejong, South Korea

**Keywords:** Lifestyle modification, Health policy, Epidemiology

## Abstract

To achieve endemic phases, repeated vaccinations are necessary. However, individuals may grapple with whether to get vaccinated due to potential side effects. When an individual is already immune due to previous infections or vaccinations, the perceived risk from vaccination is often less than the risk of infection. Yet, repeated rounds of vaccination can lead to avoidance, impeding the establishment of endemic phases. We explore this phenomenon using an individual-based Monte Carlo simulation, validating our findings with game theory. The Nash equilibrium encapsulates individuals' non-cooperative behavior, while the system's optimal value represents the societal benefits of altruistic cooperation. We define the difference between these as the price of anarchy. Our simulations reveal that the price of anarchy must fall below a threshold of 12.47 for endemic phases to be achieved in a steady state. This suggests that for a basic reproduction number of 10, a consistent vaccination rate greater than 89% is required. These findings offer new insights into vaccination-related decision-making and can inform effective strategies to tackle infectious diseases.

## Introduction

When the spread of an epidemic paralyses a society, the government adopts countermeasures, such as vaccinating people^1–3^. Vaccines not only bring benefits to vaccinated people who reduce their chances of infection from infectious diseases but may also cause side effects. A rational individual acts on the benefits and changes their strategy according to circumstances^4^. This egoistic attitude threatens public health and cannot lead to the endemic stage of infectious diseases. For example, measles and whooping cough reoccurred in the UK under the voluntary vaccination policy and eventually remained endemic^5,6^. On the other hand, COVID-19 is rapidly spreading as BA.4 and BA.5, a subvirus of Omicron, through Alpha, Beta, Gamma, and Delta. The wave of COVID-19 infections by mutations continues to nullify the success we have achieved through effective vaccine, social restriction, testing, and quarantine policies^7^. First, the level of herd immunity increases because of vaccination, showing lower severity of the virus^8^. Second, these variations interfere with the ability to recognise antibodies that threaten vaccine immunity, thereby causing reinfection^9,10^. Third, the additional vaccination rate is no longer higher than the rate of previous vaccinations. In other words, the decreasing severity and increasing reinfection rates reduce the benefits of vaccination, preventing the vaccination of reasonable individuals.

Vaccination reduces the risk of infection in people. Egoistic people try not to get vaccinated but instead rely on immunity formed from neighbouring vaccinations. They want to avoid the side effects of vaccines. For this reason, a reduction in the vaccination rate by egoistic people creates a 'social dilemma' in which vaccinations do not reduce the risk of infection but rather significantly increase the infection rate again^11–13^. Those wishing to respond to infectious diseases should understand the potential interactions in vaccine networks. In this study, we aimed to understand how individuals make vaccination decisions under the perceived risk of vaccines and diseases by reflecting on the payoff of game theory in the susceptible–infected–recovered (SIR) model.

First, we must identify the situation in which the COVID-19 pandemic reaches an endemic phase. Numerous Monte Carlo simulations can be run to mimic real-world scenarios and to obtain the corresponding results. Then, the unique uncertainty of the input parameter is reproduced by indefinitely generating unpredictable random numbers, obtaining all available results and the probability of each result. This can provide more explainable results than those of deterministic predictions. Therefore, we aimed to derive realistic scenarios by calculating the probability of all scenarios that may occur by using individual-based Monte Carlo (IBMC) simulations to predict COVID-19 diffusion patterns^14–17^. In IBMC, input parameters––including population density, personal mobility, infection, vaccination, immunity, and mortality––are used to provide accurate solutions for the model. These solutions can be directly compared to the results from the application of game theory.

By using game theory, we analysed the behaviour of those who chose vaccination and the optimal strategy to effectively achieve the benefits and those who simply wanted herd immunity. Based on the interpretation method, game theory is divided into technical and normative interpretations^18^. In technical interpretation, the theory focuses on determining the consistency with actual human behaviour, as in behaviour theory. In normative interpretation, game theory is not an approach to predict a player’s behaviour but focuses on how to behave^19^.

In terms of technical interpretation, we found evolutionarily stable strategies (ESS) by deriving the Nash equilibrium (NE) based on the basic reproduction number^20^, which is related to infectivity, by using the evolution game theory in which the ‘Vaccinated group $V$’ and ‘Unvaccinated group (free-ride to group immunity) $U$’ strategies exist. The effect of the individual's egoistic attitude on the optimal vaccination rate that can control infectious diseases was analysed. The dilemma caused the individual to adopt noncooperative egoistic behaviour, free benefits, and vaccination delay in vaccine networks, resulting in a society's vaccination rate reaching NE^21^. Society did not reach a system optimum (SO), which is an inoculation level that maximises social welfare under altruism; this occurred individuals’ egoistic behaviour^22^. The difference between NE and SO is defined as the price of anarchy (PoA)^23^. We quantified the anarchy state (i.e., the social mix) to analyse the selfish and altruistic behaviours of individuals in vaccine networks. Accordingly, this study showed that the PoA is determined by the payoff of the relative vaccinations for infection perceived by the individual^23^.

The global health crisis caused by the spread of the COVID-19 pandemic calls for significant behavioural change and places significant psychological strain on individuals^24^. Understanding behavioural epidemiology through normative interpretation of these situations can help align human behaviour with recommendations from epidemiological and public health experts^25^.

We devised a novel model to analyze vaccination behavioral dynamics, aiming to bridge the gap between individual-based model (IBM) considerations and game theoretic perspectives. The core of this approach relies on the combination of an Individual-Based Monte Carlo (IBMC) simulation and game theory. Each offers unique insights into the complex dynamics of infectious diseases and vaccination strategies. The IBMC simulation, based on non-deterministic algorithms, offers a realistic picture of individual behavior and how it influences the spread of an infectious disease, such as COVID-19. It accounts for the randomness and inherent uncertainty in the spread of the virus, including the variable basic reproduction number due to mutations.

On the other hand, our application of game theory, rooted in deterministic algorithms, provides a framework for understanding strategic decision-making around vaccination. Game theory offers insights into how varying initial parameters can yield different outcomes, which is crucial for understanding the dynamics of infectious diseases. The bridge between these two models lies in their complementary strengths. While the game theory model provides a strategic perspective on decision-making, the IBMC simulation reflects the complexity and unpredictability of real-world infection dynamics. By applying parameter values from the IBMC simulation that exhibit similar infection dynamics to the deterministic algorithms of game theory, we compensate for the deterministic model's limitations^26–28^.

Further, we analyzed the strategic changing points based on dynamic attitudinal shifts in payoff values for the vaccinations' impacts on the individual. We quantified the Price of Anarchy (PoA) values to determine whether the vaccinations lead to endemic phases as steady-state values over time^29,30^. The combination of these two models allows us to better understand the complex interplay between individual behavior, strategic decision-making, and the dynamics of infectious diseases.

## Results

### Individual-based Monte Carlo (IBMC) simulations

To realistically analyze the continually evolving pandemic, characterized by the emergence of mutant viruses and fluctuating infection rates, we employed IBMC simulations^31^. The simulation results suggest that the current coronavirus situation is challenging to control and that it takes a significant amount of time for the epidemic to reach its peak (see Fig. 8). Notably, the maximum probability of infection increases with each epidemic recurrence, showing a trend of pandemic recurrence in the probability of intermediate vaccinations. This underscores the need for a high level of vaccinations within the population to control the pandemic. Hence, the vaccination rate, which is instrumental in controlling infectious diseases, becomes a crucial factor in our analysis of individual behavior in the context of infectious diseases. However, to gain insights into controlling the infection, we need to consider a scenario where vaccinations are voluntary and the result of individual choice. It is in this context that we introduce a game theoretic model to our analysis.

The game theoretic model we adopt is directly informed by the findings from our IBMC simulations. The parameters derived from the IBMC simulations, such as the probability of infection and the vaccination rate, serve as inputs for our game theory analysis. In this way, we are able to model how individuals, acting rationally and out of self-interest, would choose to get vaccinated or not, depending on the prevailing circumstances. In doing so, we bring together the stochastic nature of the IBMC simulations and the strategic considerations of game theory to provide a more comprehensive picture of the pandemic dynamics. Thus, by combining game theory and infection dynamics, we offer a powerful analytical framework that leverages the strengths of both models—the realism of IBMC simulations and the strategic perspective of game theory. This integrated approach allows us to explore individual behavior under voluntary vaccination scenarios and provide insights into pandemic control.

### Technical interpretation of game theory

**Figure 1:**
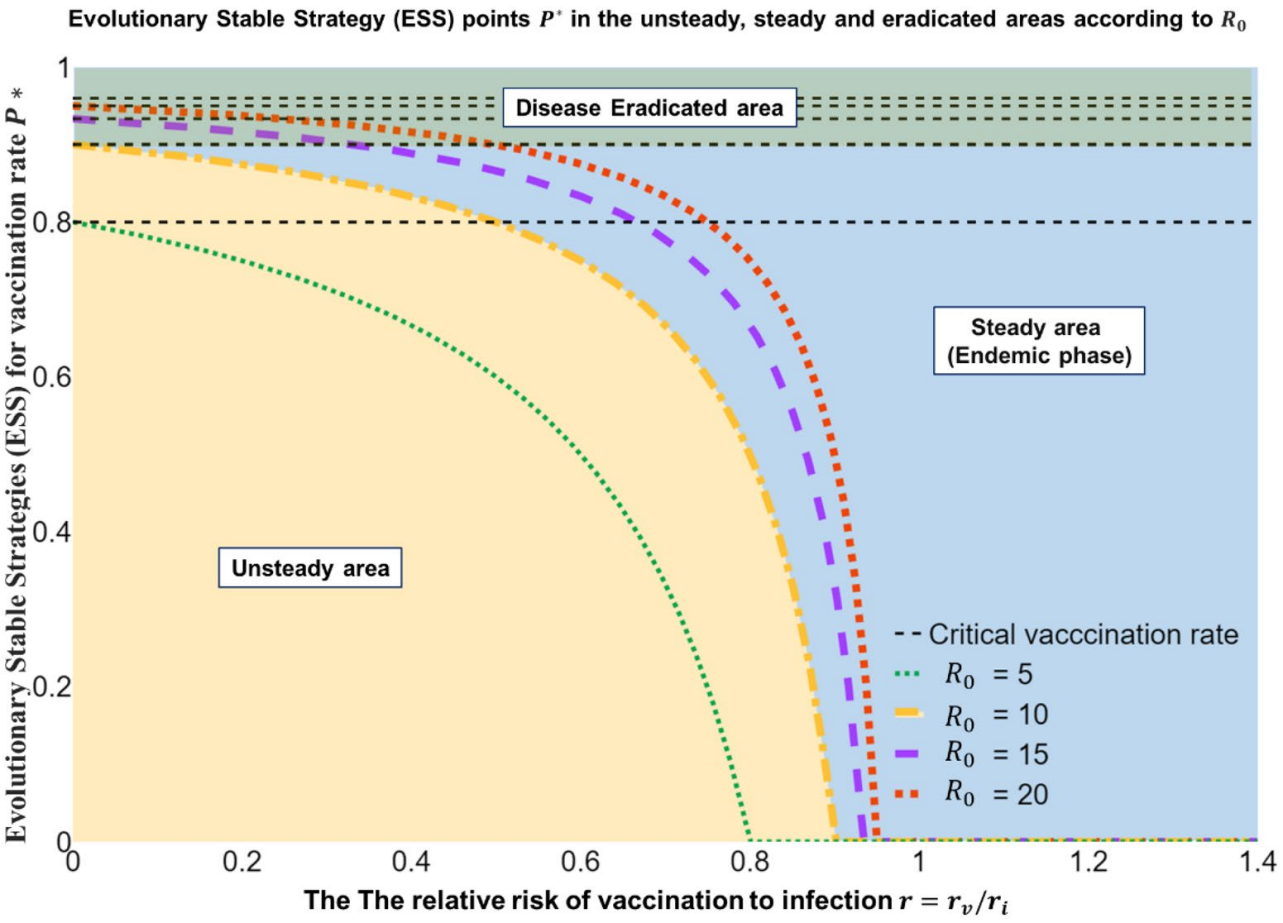
Evolutionary stable strategy (ESS) point ${P}^{*}$ in the unsteady, steady, and eradicated areas according to ${\mathcal{R}}_{0}$. If the basic reproduction number ${\mathcal{R}}_{0}$ is 10, we estimate that ${\theta }_{critic}$ is approximately 80%. We know that under the yellow dotted line is the unsteady state. The unsteady state is the state wherein society is confused because of the disease. On the other hand, over the yellow dotted line, the blue area is a steady state that is the endemic state. Here, society is stable. Finally, when reaching the black dot line, society can eradicate the disease. The dotted line ${\theta }_{crit}$ is a point where we can eradicate infection within our technical interpretation of game theory and cannot be reached in reality at ideal values. The aim of this to enter endemic phases by maintaining stability beyond the evolutionary stable strategy (ESS) ${P}^{*}$. As the basic reproduction number ${\mathcal{R}}_{0}$ increases, the stable area decreases. This is a common-sense result that the higher the infection rate is, the more difficult it is to enter endemic phases.

We found ${P}^{*},$ an ESS point that can be used to control diseases; this is like the results obtained from the IBMC simulations. This is determined by the perceived relative risk, $r$, which is defined as the relative ratio of the vaccine risk ${r}_{v}$ to the infection risk ${r}_{i}$ of the vaccine as perceived by the individual. This refinement of ${P}^{*}$ can be used to predict individual perceived risks (i.e., levels of vaccinations in voluntary vaccination policies), as shown in Fig. 1. The ${P}^{*}$ value is related to the number of basic reproduction number, ${\mathcal{R}}_{0}$. The number is an indicator of how many people can be infected when contracting an infectious disease. This is because as this number becomes larger, the infection rate increases; thus, a higher level of ${P}^{*}$ is required to cope with the increased rate. The Omicron variant has an average basic reproduction number of 9.5 and a range from 5.5 to 24^32^, so we examined ${P}^{*}$ when ${\mathcal{R}}_{0}=\{5, 10, 15, 20\}$ according to the relative risk of vaccination to infection $r$ through Fig. 1. In Table 1, the critical vaccination rate that enables disease control is ${\theta }_{crit}$, but it is almost impossible to bring $r$ close to 0 in real life. We found that vaccination alone is insufficient to control diseases in situations wherein vaccinations are voluntary^33,34^. This result is like the previously obtained IBMC simulation results.

**Table 1:** Critical vaccination rate ${{{\theta}}}_{{{c}}{{r}}{{i}}{{t}}}$ and ESS point ${{{P}}}^{{*}}$ that can eradicate diseases according to the basic reproduction number ${\mathcal{R}}_{0}$.

	${\mathcal{R}}_{0}=5$	${\mathcal{R}}_{0}=10$	${\mathcal{R}}_{0}=15$	${\mathcal{R}}_{0}=20$
${\theta }_{crit}$	0.8	0.9	0.93	0.95
${P}^{*}$($r=0.1$)	0.78	0.89	0.93	0.94

### Normative interpretation of game theory

**Figure 2:**
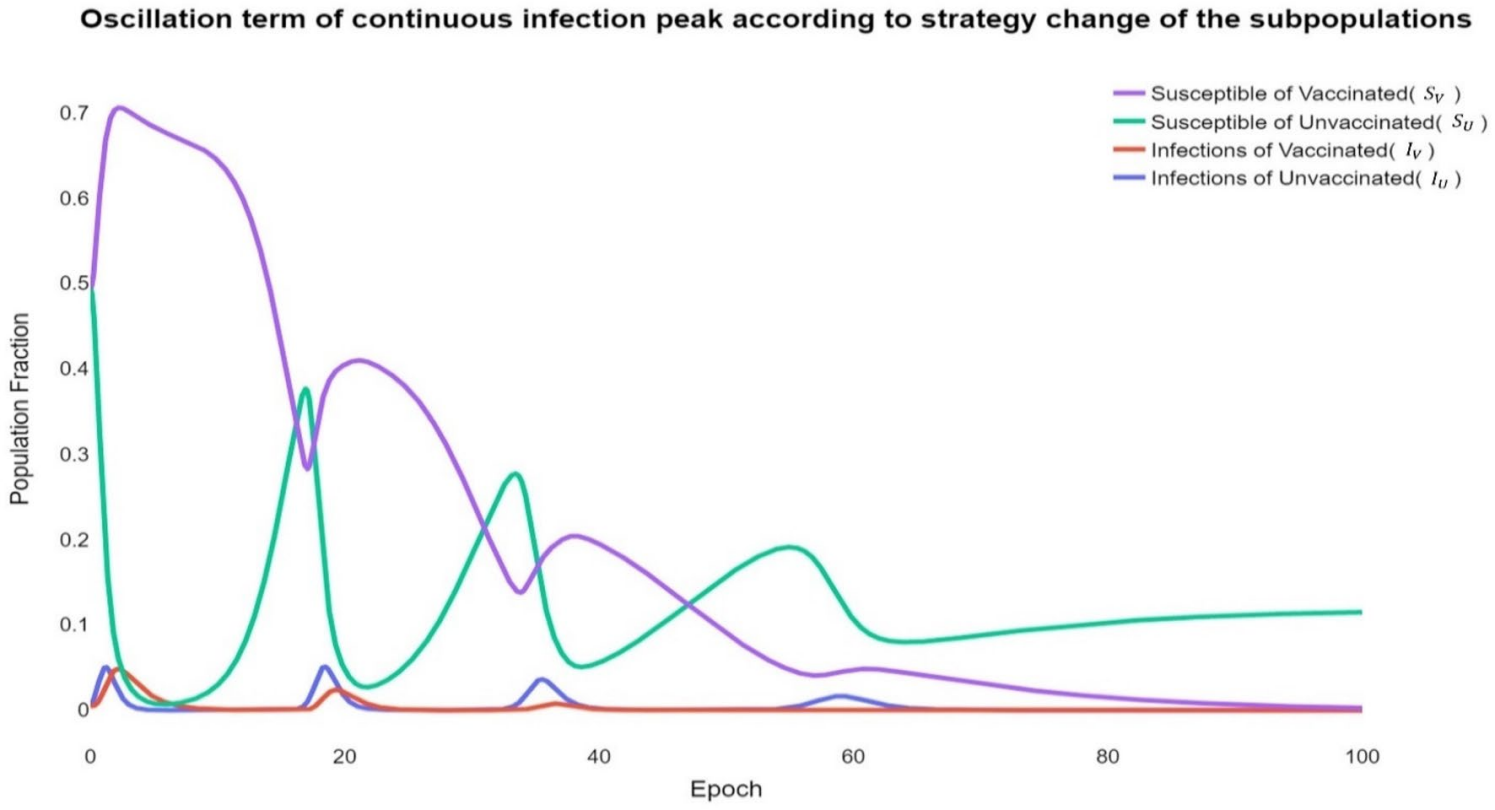
Oscillation term of continuous infection peak according to strategy change of the subpopulations ${S}_{V}, { S}_{U},{ I}_{V}, { I}_{U}$. As the infection vibration is repeated, the number of infected peaks gradually decreases. This can be seen because of the formation of group immunity through vaccination. However, as vaccinations increase, social dilemmas arise, increasing the individual reliance on vaccinations in neighbourhoods. Eventually, over time, the individual ratio of $U$ surpasses the individual ratio of $V$. Eventually, the probability of infection in $V$ decreases due to vaccination, while the individual in $U$ depends on group immunity, so it becomes higher than the probability of infection in $V$. Therefore, there are more infections in $U$ than in $V$.

The typical behavioural epidemiology involves varying populations. Although no explicit oscillation term exists in the model, dynamic scenarios in the form of repetitive waves can be analysed, as shown in Fig. 2. This phenomenon is characteristic of an observable model with various parameters^35,36^.

The underlying cause of the continuous infection peak depends on the subgeneration of each group that occurs according to strategy selection and the distribution of strategies over time. Here, $S$ means the susceptible group, and $I$ means the infected group. Moreover, $V$ and $U$ imply that each strategy is selected. In other words, ${S}_{V}, { S}_{U},{ I}_{V}, \mathrm{and} { I}_{U}$ imply that it is a subpopulation according to the $S$ and $I$ group's selection strategy $V$ or $U$.

During the early stages of infection, the vaccinated group’s payoff ${\pi }_{V}$ of the vaccinated group becomes relatively beneficial at a corresponding rate as the proportion of infected people increases. This induces the first infection peak of ${S}_{V}$. Subsequently, as most individuals cooperate with vaccinations, the proportion of infected persons and rates decrease. As $I$ approaches zero, the unvaccinated group’s payoff ${\pi }_{U}$ becomes relatively advantageous. Accordingly, the number of individuals switching from ${S}_{V}\to {S}_{U}$ increases. At the commencement of the secondary infection wave, a second peak in the infection rate inevitably occurs as an increasing number of individuals adopt the unvaccinated group strategy. A rapid increase in the unvaccinated group ratio always precedes the infection peak. In other words, the infection peak of the unvaccinated group is ahead of that of the cooperator group.

In a system comprised entirely of rational individuals, the maximum and minimum values of the strategy are consistent with ${I}{^{\prime}}$. ${I}{^{\prime}}$ is a mixed-strategy equilibrium as vaccinated and unvaccinated, and there is a probability of changing a strategy. Here, each individual selects an action based on personal observation of the same public information. This strategy assigns actions to all possible observations that individuals can perform. In game theory, if no player wants to deviate from their strategy, the strategy is called a correlated equilibrium. By correlating these strategies, the mixed strategy equilibrium (that is, NE) for all rewards in the reward vector can be achieved by using correlation strategies in noncooperative games^37^.

**Figure 3:**
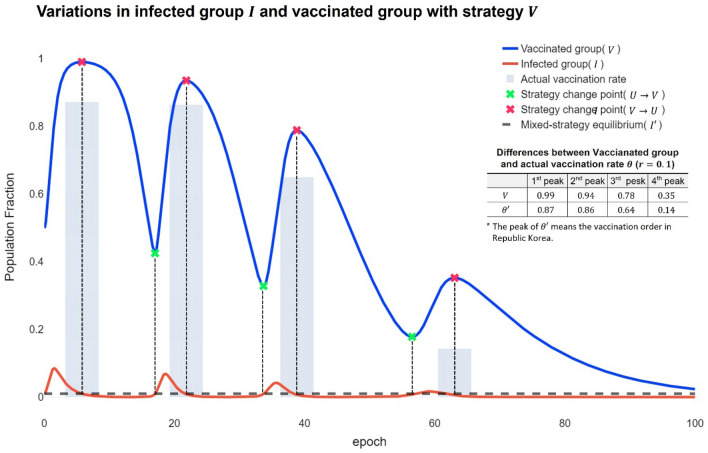
Variations in infected group $I$ and vaccinated group with strategy $V$. The blue line is the modelled value of the vaccine cooperation ratio ${{V}}$, and the sky-blue shade is the actual vaccination rate in the Republic of Korea. Higher infection rates (the highest point on the red line) reduce the relative risk of vaccination to infection ${{r}}$, which increases vaccination rates (the highest point in the blue line). The equilibrium point, the strategy change point for vaccination, occurs at convex and concave points, depending on the relative risk of vaccination to infection. In the first left, ${{\theta}}$ cannot be 1 because ${{{P}}}^{{*}}$ does not exceed ${{{\theta}}}_{{{c}}{{r}}{{i}}{{t}}}$, even though the group ${{V}}$ almost reaches 1. This leads to a repeated wave of infection. The intervals between the modelled value and the real value indicate the difference between the endemic phases and reality.

When individuals choose the $V$ strategy with a probability of ${P}^{*},$ the numerical analysis of the ordinary differential equation integral shows that even when applying the Fermi strategy probability, it is consistent with such predictions (Fig. 3). This analysis is consistent with all the analysed values for ${r}_{i}$, ${r}_{v}$, and the infection rate in the delayer ${\beta }_{U}$. Moreover, the peak of inflection always occurred between the maximum and minimum values of $V$.

In the 1st peak of $V$, 0.99 of the population adopts the $V$ strategy. In a situation where ${\mathcal{R}}_{0}=10$ and $r=0.1$ of Omicron, ${P}^{*}$ is 0.88. Then, the vaccination rate of the population does not reach ${\theta }_{crit}$ according to Eq. (6) in the “Methods” section. Therefore, from the first peak, we do not expect an eradication of Omicron. The actual vaccination rate $\theta ^{\prime}$ in the Republic of Korea is also close to 0.88, but it can be seen that ${\theta }_{crit}$ has not been reached. In addition, as the importance of vaccination decreases due to repeated vaccinations, $r$ eventually decreases, indicating that the number of individuals adopting the $V$ strategy decreases. This is consistent with the result of $\theta ^{\prime}$. In other words, the vaccination rate in the population $\theta$ eventually decreases as the number of individuals adopting the $V$ strategy decreases. In other words, these scenarios suggest that the previous pandemic was like the expected scenario of the recurrent infection wave^38,39^. such as infectious wave behaviour. Here, the social dilemma of vaccination is also observed. This is because the number of individuals who want to free-ride herd immunity increases^40,41^ due to repeated vaccinations. This allows us to expect Omicron to eventually move towards the endemic phase.

The quantity $\zeta$ denotes the coupling constant between the epidemiology of diseases and the evolutionary game dynamics. It defines how rapidly a population can respond to new information regarding the current state of a disease. Therefore, if the value of $\zeta$ increases, the response speed changes; hence, strategy changes frequently occur. Therefore, if $\zeta$ increases, the individual responds quickly to new information, increasing the likelihood of adopting a $V$ strategy. In other words, the more individuals immediately respond to information and cooperate, the greater the peak of the infection is divided, reducing the maximum peak of the infection that can greatly threaten society.

**Figure 4:**
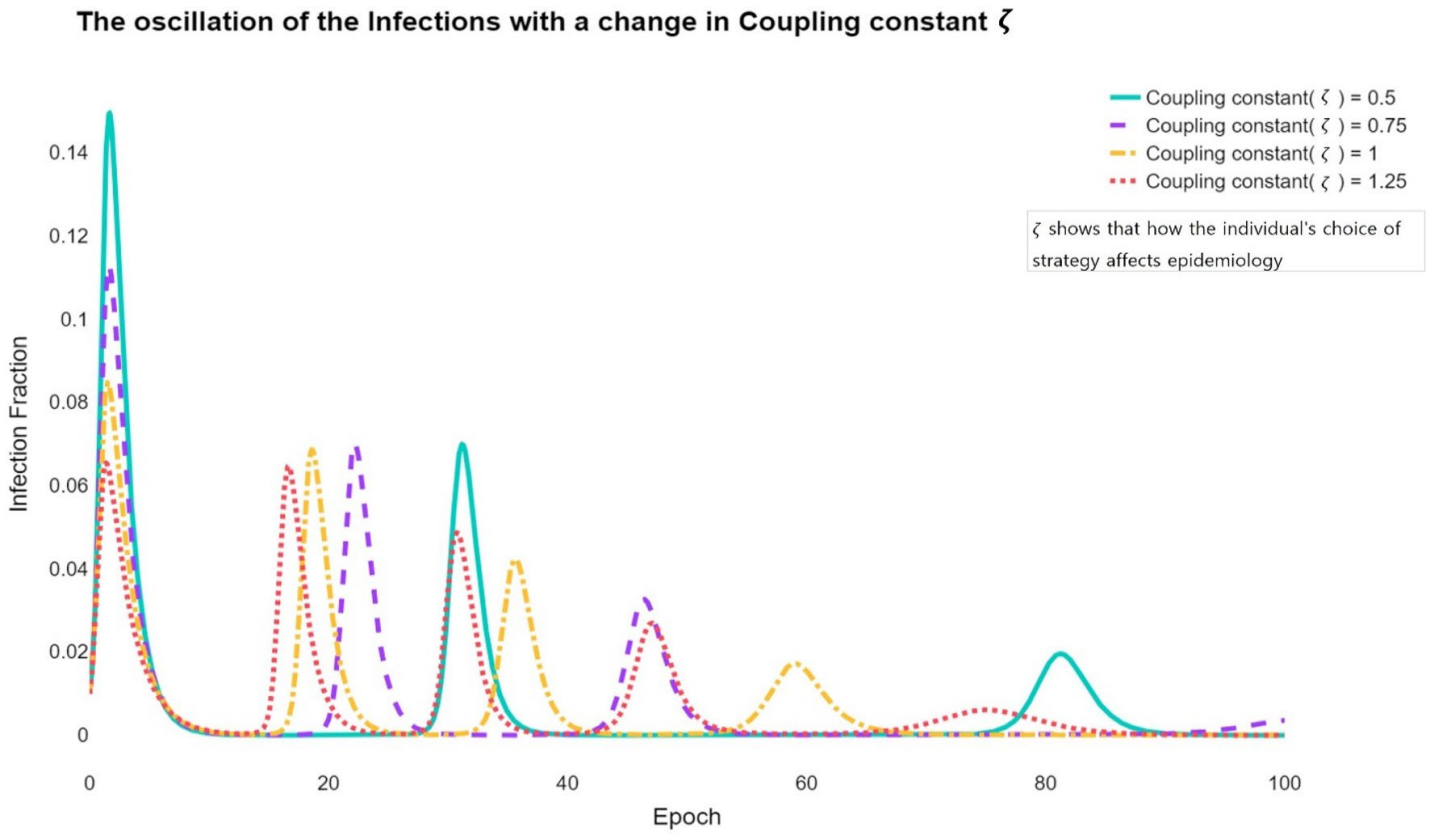
The oscillation of the $I$ peak with a change in $\zeta$. The behavioural epidemiology of $I$ changes with $\zeta$ oscillations occur frequently as $\zeta$ increases. This induces more oscillations in the entire population. However, the change in $\zeta$ does not influence $I$. This parameter does not ultimately fluctuate the size of the infection but rather disperses the size to make the oscillations more frequent.

Strategies $V$ and $U$ in our model can be divided into vaccination cooperating and noncooperating groups, respectively. Figure 4 shows how the behavioural epidemiology that an individual chooses $V$ or $U$ changes with the speeds at which an individual can access and respond to the information.

If the vaccination rate $\theta$ is close to ${P}^{*}$, $I$ can be expected to control the disease by reaching SO. This implies that the infection rate of the vaccinated group ${\beta }_{V}$ is the same as that of the unvaccinated group ${\beta }_{U}$, thus indicating convergence and consequent control of the disease. However, we found that it is difficult to reach SO based on voluntary vaccinations via technical interventions in the game theory model (Fig. 3). This is because when $V$ increases and reaches its peaks, $U$ power is adopted to rely on individual herd immunity. Finally, more individuals adopt the $U$ strategy, resulting in another spread of infection. Eventually, the vaccination rate in society reaches NE, and infections continue to occur. This could be attributed to a social dilemma regarding vaccination caused by the ‘externality’ effect of the vaccinated individual. This observed counterintuition is equivalent to the well-known Braess’ paradox in the traffic flow problem, in which more roads could lead to more severe traffic congestion. We quantified the PoA (price of anarchy) and the ratio of SO to NE to analyse the individual's egoistic and altruistic attitudinal behaviour in these vaccination network problems as Eq. (1)^42^:

1 $${{PoA}} = \frac{{\sum {{t}}_{{{{ij}}}} \left( {{{f}}_{{{{ij}}}}^{{{{NE}}}} } \right){{f}}_{{{{ij}}}}^{{{{NE}}}} }}{{\sum {{t}}_{{{{ij}}}} \left( {{{f}}_{{{{ij}}}}^{{{{SO}}}} } \right){{f}}_{{{{ij}}}}^{{{{SO}}}} }} = \frac{{{{I}}_{{{{max}}}}^{{{{NE}}}} }}{{{{I}}_{{{{max}}}}^{{{{SO}}}} }}$$

**Figure 5:**
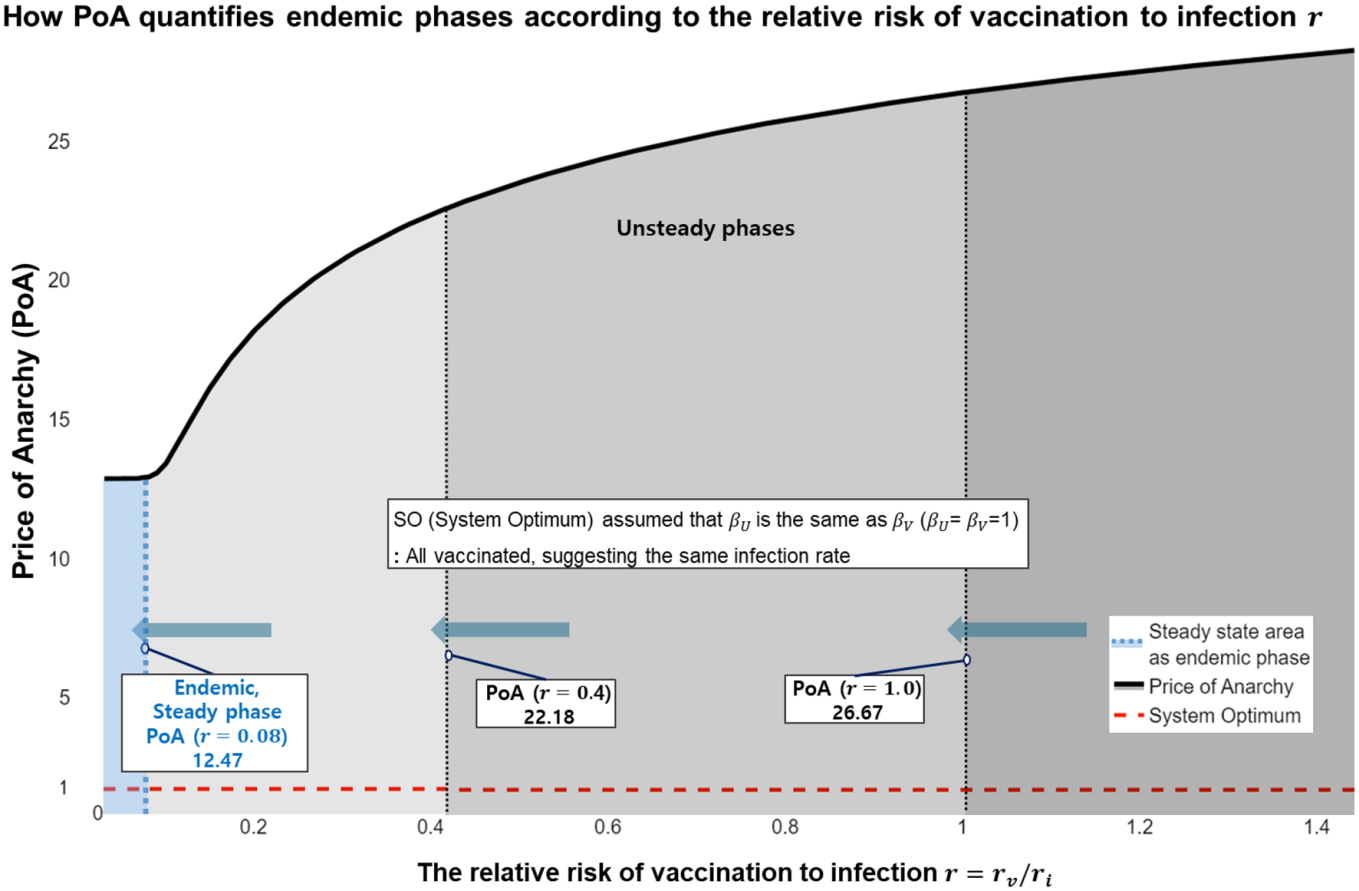
Changes in the PoA and how to lead endemic phases according to the $r$. The PoA shows the difference between the end value, the system optimum (SO), and the realistic value, the Nash equilibrium (NE), which can be obtained from the ideal value ${\theta }_{crit}$. Our objective is to reduce the gap between SO and NE and induce endemic phases. In this study, we obtained the steady state of PoA to find the endemic phases and then calculated PoA to make the vaccination policy effective. PoA is affected by the degree to which vaccination is dangerous compared to infection, and the relative risk of vaccination to infection is $r={ r}_{v}/{r}_{i}$. The lower the $r$, the higher the vaccination rate, which is the process of reaching an evolutionarily stable state. The rate converges when $r$ is less than or equal to approximately 0.08 and moves on to the endemic phases. PoA decreases from 28 to 12.47 according to $r$. In this case, if ${\mathcal{R}}_{0}=10$, the vaccination probability ${P}^{*}$ (evolutionary stable strategy (ESS)) required is 89% (see Table 1). This result indicates that increasing the vaccination rate by lowering the relative risk of vaccination to infection $r$ as a positive policy for vaccines can lead to social stability that can lower PoA and further induce the steady state to reach the endemic phases.

In transportation, the PoA is calculated to suggest plans for smooth transportation by identifying the travel time delay caused by the egoistic behaviour of individual passengers in the transportation network^42^. In this case, ${f}_{ij}^{NE}$ is the traffic volume in NE, and ${f}_{ij}^{SO}$ is the traffic volume in SO. ${t}_{ij}({f}_{ij})$ is the travel time according to each traffic volume ${f}_{ij}$. Thus, $\sum {t}_{ij}({f}_{ij}){f}_{ij}$ is calculated, and the travel time required to pass the link is converted into a cost to determine the network efficiency. If PoA $>1$, the toll cost for NE is higher than that of SO, indicating an inefficient network. In other words, the closer the PoA is to one, the greater the network can move towards maximising social welfare^23^. The most critical feature in epidemiology is to recommend a pre-emptive plan according to ${I}_{max}$. Therefore, we calculated the PoA based on the ratio of ${I}_{max}$ in NE to ${I}_{max}$ in SO to obtain the index that can determine ${I}_{max}$ according to the individual's behaviour. Through this PoA concept, we can quantify the extent to which social inefficiency and losses were caused by egoism compared to when the overall gain of society was maximised. The quantification of the PoA was to effectively manage epidemiology in the vaccination network problem, as shown in Fig. 5.

The graph for ${\beta }_{U}={\beta }_{V}=1$ presented in Fig. 5 illustrates the SO results, in which the number of infected people converges to zero. However, the reality is that SO is not reached because of a dilemma, resulting in ${\beta }_{U}>{\beta }_{V}$. Therefore, the results shown in Fig. 5 are obtained, suggesting that the PoA is not close to 1 with increasing ${I}_{max}$. Here, decreases in $r$ lead to a reduction in PoA and ${I}_{max}$, showing a more cooperative attitude towards vaccinations. As a result, ${I}_{max}$ decreases. Figure 1, Table 1, and Fig. 3 show that Omicron is becoming endemic. However, as shown in Fig. 3, repeated inoculations increase $r$, which reduces the proportion of individuals taking the $V$ strategy, and from Fig. 5, we can see that the increase in $r$ moves towards increasing PoA. In other words, when r increases, society goes to an unsteady situation, not a steady endemic, and then ${I}_{max}$ does not converge but rather increases. At this time, when $r=0.08$ is reached, the state of the steady endemic may be switched. However, according to Fig. 3, the proportion of the population adopting the $V$ strategy in the phases of repeated inoculations decreases.

Thus, we must maintain a vaccination probability above ${P}^{*}$ and increase $V$ in the population to maintain steady endemicity. Therefore, we should proceed to the steady endemic state by reducing the relative risk of vaccination to infection $r$ in Fig. 5 to increase the proportion of the $V$. This should reduce PoA to maintain a stable society from infection.

## Discussion

First, the results of technical interpretation can predict the level of vaccination rates that can control infectious diseases in society based on the relative risk of vaccination to infection, as perceived by individuals. We found that the vaccination rate increased as the basic reproduction number increased. Currently, the infection reproduction index of COVID-19 is increasing owing to the continuing occurrence of mutant viruses^31^. In addition, the importance of vaccines is decreasing owing to repeated vaccinations. This implies that the relative risk may increase, resulting in a situation in which society may eventually achieve an endemic phase.

In addition, we found from normative interpretations that selfish individuals switch individual strategies according to the average expected payoff that varies with vaccination rates. When an individual's cognitive risk is less than 1, the individual is reluctant to adopt a vaccination strategy and wants to ride free on the herd immunity generated by vaccinations in the neighbourhood. This can lower the vaccination rate and leads to infection resurgence. This is a social dilemma phenomenon of vaccines stemming from individual "self-interest," which can be interpreted as the cause of the oscillations in the infection wave in Monte Carlo simulations. As a result, in a situation wherein infectious diseases are spreading, if certain individuals implicitly refuse to receive the promised vaccination, a certain amount of anarchy occurs in society. The question arises as to who the victims of the anarchy are. This is because the subject who suffers the consequences is not only those who violate the rules but the entire society; this causes a social dilemma. To present countermeasures to minimise social dilemmas, we quantified it as the price of anarchy.

As indicated by the results, it is relatively difficult for a society composed of rational individuals to overcome diseases. Therefore, we must shift our policy towards reducing the amplitude of the epidemic. $\theta$ denotes the probability of the vaccination rate in population $\varepsilon$. Compared to $U$, $V$ indicates vaccinating without showing a delay in the vaccination policy. The higher the probability is, the higher the $\theta$ value and the closer it may be to ${\theta }_{crit}$ to facilitate disease control. It is difficult to expect ${P}^{*}$ beyond ${\theta }_{crit}$ owing to social dilemmas arising from technical interpretations of the Monte Carlo simulations and game theory. However, to lower the PoA, it is important that we promote the vaccination rate of the real population to be above ${P}^{*}$. If the vaccination rate of the population exceeds ${P}^{*}$, a society can expect a more stable situation from infectious diseases because of the adoption of an ESS. It is crucial to increase the risk perception of infection to reduce the relative cognitive risk, thereby lowering the risk of vaccines^43^. In addition, we found through normative interpretation that the faster the response to new information, the more dispersed the infection peak, thereby reducing the size of the maximum infection. Thus, we quantified the behaviour of an individual in vaccination through the results from applying game theory in situations wherein infectious diseases tend towards the endemic phase. We found that it is important to lower the risk of vaccines relatively quickly and to vaccinate individuals to reduce the social dilemma.

This study adopted a two-pronged approach to simulate the spread of infectious diseases and understand individual behaviour in this context. Firstly, we used Monte Carlo simulations to model the stochastic nature of disease spread, taking into account the continuous emergence of mutant viruses and random changes in infection rates. Secondly, we employed game theory to analyse the strategic choices individuals make regarding vaccination, based on their perceptions of relative risks and benefits. The Monte Carlo simulations generated a range of possible outcomes, providing us with a set of key parameters that informed our game theory model. These parameters, such as the probability of infection and the vaccination rate, represented the 'game's' current state, upon which individuals based their strategic choices. In the game theory model, individuals, assumed to act rationally and in self-interest, decided whether to vaccinate or not based on these parameters. The dynamic interplay between individual decisions and the evolving state of the game led to different vaccination rates, which in turn influenced the course of the disease spread. By integrating these two models, we were able to overcome the limitations of deterministic algorithms and provide a more comprehensive picture of the infectious disease spread. This combined approach allowed us to mathematically quantify social phenomena caused by individual selfishness, a crucial aspect in infectious epidemiology. We identified a steady state and targeted vaccination rates for populations above ${P}^{*}$, providing valuable insights for promoting vaccination rates in societies battling infectious diseases. Our findings suggest strategic directions for vaccine strategies to become an evolutionarily stable strategy (ESS).

Despite our efforts to combine individual-based modelling and game theory, one limitation of our study lies in the assumption of rational behaviour among individuals. In reality, individuals' decisions about vaccination can be influenced by a variety of factors, including misinformation, personal beliefs, and societal pressures, which our model does not fully account for. Furthermore, the constant emergence of new virus variants and changes in societal behaviour could affect the efficacy of vaccination strategies, adding another layer of complexity not fully captured in our current model. Future research should therefore focus on incorporating these dynamic factors into the model to better understand and predict the course of infectious diseases in real-world scenarios.

## Methods

### IBMC simulations

In this study, the IBMC system consisted of $N$ individuals randomly located in a square plane of ${L}^{2}$, corresponding to the population density, $\rho =\frac{N}{{L}^{2}}$. Individuals represented as structureless points in the model were infected with probability ${p}_{infect}$ when they were within an infectious distance, ${r}_{infect}$, from infected individuals. Infected individuals had incubation time ${t}_{incubation}$, after which they faced two fates: immune after ${t}_{infect-immune}$ or dead with probability ${p}_{fatal}$ during the infected period, ${t}_{infect}$. Immunisation is effective during ${t}_{infect-immune}$. Individuals can also be vaccinated with probability ${p}_{vacc}$ and become immune from ${t}_{vacc-start}$ after vaccination until ${t}_{vacc-start}+{t}_{vacc-immune}$, where ${t}_{vacc-immune}$ is the effective immunisation period. Table 2 lists the parameter values used in this study.

**Table 2:** Parameters used in Individual-Based Monte Carlo (IBMC) simulations.

Parameter	Value	Meaning
$N$	1000	Number of individuals
$L$	246 m	System size
$\rho =N/{L}^{2}$	0.165	Population density
${r}_{infect}$	1 m	Infectious distance
${p}_{infect}$	1.8%	Probability of infection
${p}_{fatal}$	0.8%	Probability of fatality
${p}_{vacc}$	0–80%	Probability of vaccination
${t}_{incubation}$	7.74 $\pm$ 4.39 days	Incubation period of COVID-19
${t}_{infect}$	14 $\pm$ 7 days	Infectious period after incubation period
${t}_{vacc}$	14 $\pm$ 7 days	Time it takes for immunisation after vaccination
${t}_{infect-immune}$	180 days$\pm 30$	Immunisation period after infection
${t}_{vacc-immune}$	180 days$\pm 30$	Immunisation period after vaccination
$\tau$	0.001 day	MC time step
$\Delta R$	1.0 m	MC step size

The population density, $\rho$, of $0.165 {\mathrm{m}}^{-2}$, which is ten times as high as that of Seoul, South Korea, was used to mimic the human-congested area, and the infectious distance when unmasked was approximated as 1 m. In addition, probabilities of infection and fatality were set to 1.8% and 0.8% based on the real data from the Centers for Disease Control and Prevention in the Republic of Korea (CDCP)^44^, respectively, from which the incubation period (7.74 $\pm$ 4.39 days) of COVID-19 was also taken. On the other hand, both the infectious period after the incubation period and the time it takes for immunisation after vaccination were approximately set to 14 $\pm$ 7 days because the reported values are typically one ton two weeks. In the same spirit, we set immunisation periods from both infection and vaccination to 6 $\pm$ 1 months because the recommended vaccination period is reported to be approximately five to six months.

Even though the parameters used in the IBMC simulations are arbitrary, the presence of the recurrent pattern observed in the simulations is rather insensitive to the parameter space. For example, the population ratios of various types of individuals when $\rho =0.08 {\mathrm{m}}^{-2}$ and ${p}_{vacc}=0$ are shown in Supplementary Fig. 1.

Initially, $N$ healthy individuals were randomly generated in the square plane of ${L}^{2}$, and one individual was selected as infected. Healthy individuals were then randomly vaccinated with a probability ${p}_{vacc}$. Each individual, except for dead individuals, randomly moves with diffusion coefficient $D=\frac{({\Delta R)}^{2}}{4\tau }$ according to the Einstein relation^45^, where $\Delta R$ and $\tau$ are the step size and time step, respectively. The simulation adopts the periodic boundary condition in both the x- and y-directions to prevent the wall effect at the edges^45^. The results are the averages and one standard deviation of 10 independent IBMC simulations.

**Figure 6:**
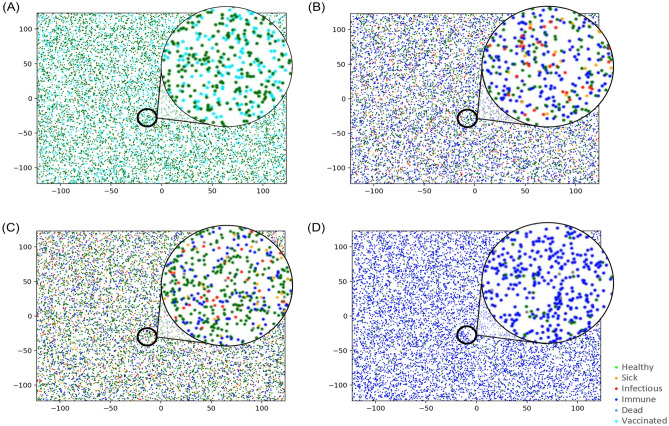
IBMC simulation snapshots taken at (**a**) t = 0, (**b**) t = 340, (**c**) t = 760, and (**d**) t = 1130 days. At (**a**), 40% were vaccinated (cyan). The 2nd and 3rd outbreaks took place at (**b**) and (**c**), and the pandemic ended at (**d**), where approximately 90% of individuals were immune (blue).

**Figure 7:**
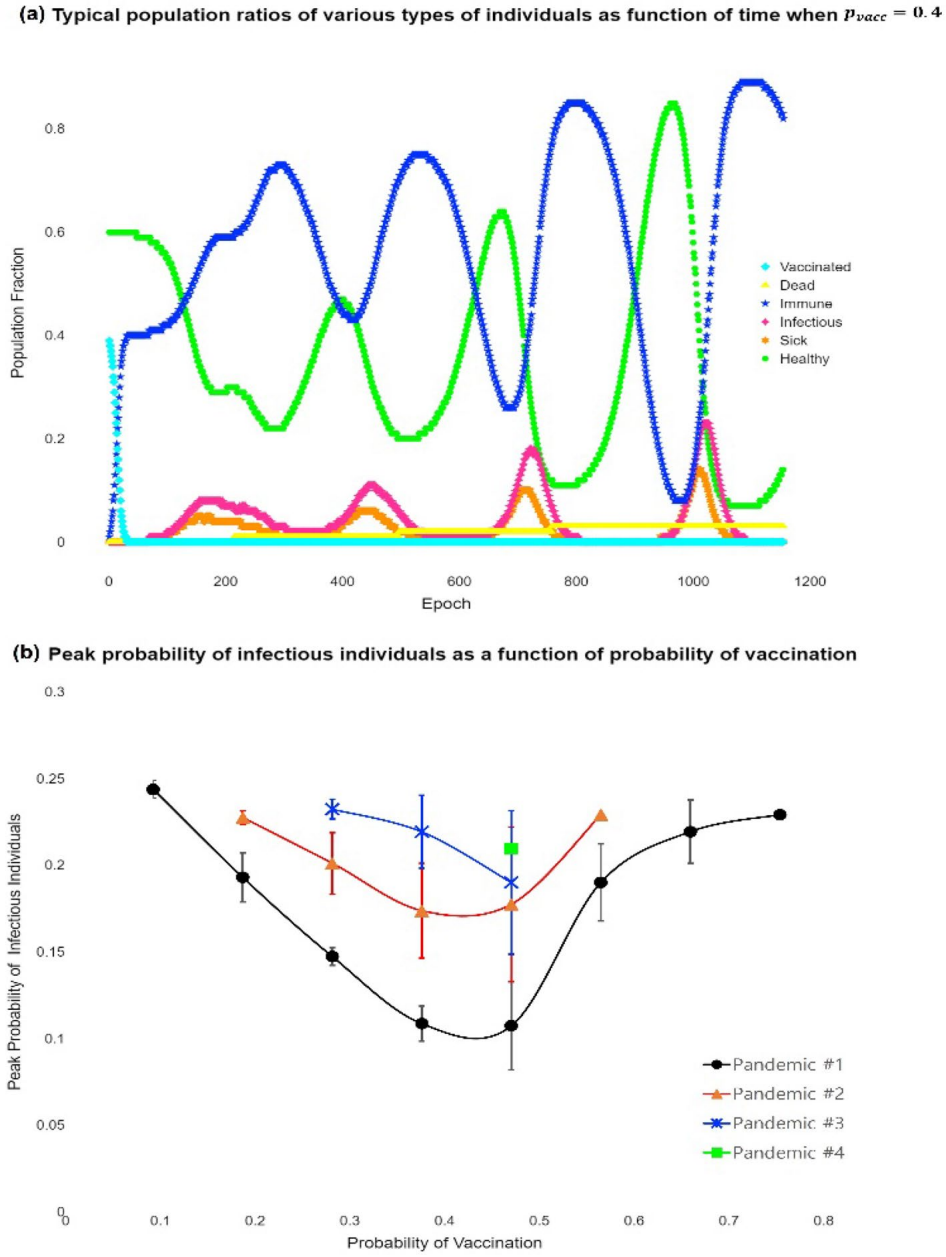
(**a**) Typical population ratios of various types of individuals as a function of time when ${p}_{vacc}=0.4.$ Healthy, sick but not infectious, infectious, immune, dead, and vaccinated individuals are represented by green, orange, red, blue, grey, and cyan, respectively. (**b**) Peak probability of infectious individuals as a function of the probability of vaccination ${p}_{vacc}$.

Figure 6 presents the IBMC simulation results. Figures 6 and 7a show the typical time progression of various types of individuals (healthy, sick but not infectious, infectious, immune, dead, and vaccinated) when the probability of vaccination ${(p}_{vacc})$ is 0.4 (the corresponding animation is given in Supplementary Fig. 1). Supplementary Fig. 1: The animation of the IBMC simulation when ${p}_{vacc}=0.4$ is presented in which green, organ, red, blue, grey, and cyan colours correspond to healthy, sick but not infectious, infectious, immune, dead, and vaccinated individuals, respectively.

The following points are of significance: first, the pandemic occurred recurrently, which implies that it is challenging to control COVID-19. Second, it takes time for the pandemic to bloom. In this case, it took approximately 200 days for the pandemic to reach its peak. The pandemic had a long incubation period, considering that there was only one infected individual initially. Third, mass infection boosts mass immunisation, reducing the number of infected people. Finally, the peak probability of infection increased as the pandemic recurred, which is shown in Fig. 7b, in which the peak probability of infectious individuals is plotted as a function of the probability of vaccination. The pandemic continues until the peak probability of infectious individuals reaches at least 25%. Moreover, the recurrent trend of the pandemic appears only in the intermediate vaccination probability ($p_{vacc} = 0.1 - 0.5$). This indicates that a high mass vaccination rate is required to end the pandemic. It should also be noted that the case for ${p}_{vacc}\ge 0.8$ does not suffer from the pandemic at all.

After obtaining the output from our IBMC simulations, we transitioned to the game theory model by considering the individual behaviors observed during the simulations. Specifically, we treated the individual's decision to get vaccinated as a strategic choice in the context of game theory. Each individual's payoff, in this case, can be understood as the personal benefit derived from either getting vaccinated or refusing vaccination, given the vaccination statuses of other individuals in the population. This is where the concept of an Evolutionarily Stable Strategy (ESS) becomes relevant. An ESS is a strategy that, if adopted by a population in a game, cannot be invaded by any alternative strategy that is initially rare. It is 'stable' in the sense that small deviations from it will be reabsorbed into it. We considered an individual's decision to get vaccinated as an ESS under certain conditions. We approximated the parameters of the game theory model based on the results from the IBMC simulations. The transition from the IBMC simulations to the game theory model involved mapping the individual behaviors and interactions observed in the simulations to the strategic choices and payoffs in the game theory model. This allowed us to capture the complex, individual-level dynamics of infectious disease spread in a theoretically rigorous framework.

### Evolutionary game theory

First, the definitions of the indices, the parameters and the variables used in this research are listed in Table 3.

**Table 3:** Parameters used in evolutionary game theory and the SIR model.

Parameter	Meaning
$P$	One individual’s probability of vaccination
${P}^{*}$	Evolutionary stable strategies point
$Q$	Another individual’s probability of vaccination
$V$	Vaccinated group
$U$	Unvaccinated group
${r}_{v}$	Perceived risk of vaccination
${r}_{i}$	Perceived risk of infection
$r$	Perceived relative risk
$\beta$	Average infection rate
${\beta }_{V}$	Infection of vaccinated group
${\beta }_{U}$	Infection of unvaccinated group
${\beta }_{c}$	Cross-infection rate ($V\leftrightarrow U$)
$c$	Crossing parameters between $V$ and $U$ groups
$\gamma$	Average recovery period
$\mu$	Average birth rate
$\varepsilon$	Population
${\pi }_{V}$	Vaccinated group’s payoff
${\pi }_{U}$	Unvaccinated group’s payoff
$E$	Average expected payoff
${E}_{V}$	Expected payoff to individuals playing $V$
${E}_{U}$	Expected payoff to individuals playing $U$
$\Delta E$	Scale of attraction from $U$ to $V$
$\theta$	Vaccination rate
${\theta }_{crit}$	Critical vaccination rate
$S$	Susceptible group
${S}_{V}$	Sensitivity of vaccinated group
${S}_{U}$	Sensitivity of unvaccinated group
$I$	Infected group
${I}_{V}$	Infection of vaccinated group
${I}_{U}$	Infection of unvaccinated group
$I^{\prime}$	Mixed-strategy equilibrium, strategy change point
$R$	Recovered group
${R}_{V}$	Recovered of the vaccinated group
${R}_{U}$	Recovered of the unvaccinated group
$\delta$	Epoch
$t$	Time
$f$	Part of the average life span
${\mathcal{R}}_{0}$	Basic reproduction number
$\Theta \left({\pi }_{i}, {\pi }_{j}\right)$	The probability that an individual who adopts i changes strategy to j
${\Phi }_{S}$	Strategy conversion rate of the susceptible group
${\Phi }_{S}$	Strategy conversion rate of the infected group
$\zeta$	Coupling constant between the epidemiology of diseases and the evolutionary game dynamics
$k$	Irrationality of changing these strategies

We proposed a model with ‘Vaccinated group $V$*’* and ‘Unvaccinated group (delay vaccination to get free rides on herd immunity) $U$*’*. In game theory, the former strategy can be interpreted as a form of cooperation, and the latter as selfish behaviour or betrayal. The individual establishes a strategy based on the perceived risk of the current behaviour^46,47^. The individual perceived risks of the vaccine and infection are denoted by ${r}_{v}$ and ${r}_{i}$, respectively. The vaccinated group’s payoff ${\pi }_{V}$ is defined as ${-r}_{v}$ (Eq. (2)). An unvaccinated group’s payoff ${\pi }_{U}$ is defined as ${r}_{i}{\beta }_{U}I$ because it can be assumed that they act according to the infection rate of the unvaccinated group ${\beta }_{U}$ and the proportion of infected people in the group (Eq. (3)). We assumed that all individuals received the same information to simplify the model, and this information was accepted completely to perceive the risk^48^.

2 $${{{\pi}}}_{{{V}}}=-{{{r}}}_{{{v}}}$$

3 $${{{\pi}}}_{{{U}}}=-{{{r}}}_{{{i}}}{{{\beta}}}_{{{U}}}{{I}}$$

We suppose that the probability that the individual is vaccinated is $P$. Since the only actions available to the individual are vaccination and no vaccination, the probability of choosing unvaccinated is $1-P$. If the individual uses a mixed strategy of ‘choose vaccination with the probability of $P$ and choose unvaccinated with the probability of the remaining $1-P$*’*, the average expected payoff of the Individual is described as Eq. (4).

4 $${{E}}={{P}}{{{\pi}}}_{{{V}}}+\left(1-{{P}}\right){{{\pi}}}_{{{U}}}={{P}}\left({{{r}}}_{{{i}}}{{{\beta}}}_{{{U}}}{{I}}-{{{r}}}_{{{v}}}\right)-{{{r}}}_{{{i}}}{{{\beta}}}_{{{U}}}{{I}}$$

Here, because the individual acts based on the relative perception of vaccines and infection risks, $r$, it is possible to simplify and represent variables by using $r={r}_{v}/{r}_{i}$ (Eq. (5)).

5 $${{E}}=-{{r}}{{P}}-{{{\beta}}}_{{{U}}}{{I}}(1-{{P}})$$

We attempted to identify which strategies can be adopted. If the majority of the population adopts strategy $V$ and an entity adopting another strategy $U$ always performs lower than that of an entity adopting $V$, then $V$ is the best response strategy. If this is true for any $V\ne U$, V is called the ESS. If $V$ is the evolutionary stable strategy and everyone is currently playing $V$, no one should change their strategy. We suppose $Q$ is the probability that another individual chooses to vaccinate when one individual is agonised about vaccination with the probability of $P$. Then, whether an individual selects $V$ or $U$ depends on the probability $Q$ that another individual adopts $V$. If this is expanded to the population $\varepsilon (0\le \varepsilon \le 1)$, the vaccination rate at the population $\theta$ is described as Eq. (6).

6 $${{\theta}}={{\varepsilon}}{{P}}+(1-{{\varepsilon}}){{Q}}$$

The expected payoff to individuals playing $V$ is given as Eq. (7).

7 $${{{E}}}_{{{V}}}={{E}}({{V}},{{\varepsilon}}{{P}}+(1-{{\varepsilon}}){{Q}})$$

whereas the expected payoff to individuals playing $U$ is given as Eq. (8).

8 $${{{E}}}_{{{U}}}={{E}}\left({{U}},{{\varepsilon}}{{P}}+\left(1-{{\varepsilon}}\right){{Q}}\right)$$

The payoff gains to an individual playing $\theta$ in such a population are given as Eq. (9).

9 $${{\Delta}}{{E}}={{{E}}}_{{{V}}}-{{{E}}}_{{{U}}}=\left({{{\beta}}}_{{{U}}}{{I}}-{{r}}\right)\left({{V}}-{{U}}\right)$$

$\Delta E$ is represented by a scale of attraction from $U$ to $V$. Equation (9) shows that the best response $\varepsilon$ depends on $r$. Then, depending on the given $r$, there is a unique strategy $P={P}^{*}(Q \ne P)$ that satisfies the best response in $\varepsilon$. That is, the unique strategy ${P}^{*}$ under the condition that $\Delta E$ is positive means NE, and if the $V$ strategy is adopted with a probability close to $P$, it is an ESS^49^.

### Technical interpretation of game theory

**Figure 8:**
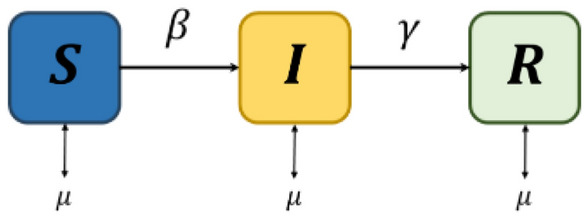
Behavioural epidemiology model. $S,I,$ and $R$, generalised when using the SIR vaccination model. The model represents a state of dynamics. $S$ represents the susceptible group with a possibility of infection. $I$ represents the infected group. $R$ refers to the recovered group in which immunity to infection is generated. $\beta$ is the average infection rate that occurs in the population. The incidence of infected people varies with $\beta$. $\gamma$ is the average recovery period related to the conversion of the *I* individual to the *R* group. The average birth and mortality rate $\mu$ represents the number of births and deaths within the population.

In terms of technical interpretation, we analysed the SIR model. The model is defined as the rate of change in the population proportion in each compartment. Generalising the results of the proposed model when using the SIR vaccination model enables the population compartment to be expressed as shown in Fig. 8 and Eqs. (10, 11, 12) below.

10 $$\frac{{{d}}{{s}}}{{{d}}{{t}}}={{\mu}}\left(1-{{\theta}}\right)-{{\beta}}{{S}}{{I}}-{{\mu}}{{S}}$$

11 $$\frac{{{d}}{{I}}}{{{d}}{{t}}}={{\beta}}{{S}}{{I}}-{{\gamma}}{{I}}-{{\mu}}{{I}}$$

12 $$\frac{{{d}}{{R}}}{{{d}}{{t}}}={{\mu}}{{\theta}}+{{\gamma}}{{I}}-{{\mu}}{{R}}$$

where $\mu$ is the average birth rate, $\beta$ is the average infection rate, $\upgamma$ is the average recovery period, and $\varepsilon$ is the population. After reaching a dynamic steady state, the vaccine coverage level in the population equals the uptake level. Because we focused on the steady-state solution of the model, our notation $\theta$ for vaccine uptake is consistent with the payoffs in game theory as Eq. (6) in our notation. In the established SIR model, the third equation is redundant because $S+I+R=1$. Therefore, we can define $S$ and $I$ as Eqs. (13) and (14)^50^.

13 $$\frac{{{d}}{S}}{{{d}}{{\delta}}}={{f}}\left(1-{{\theta}}\right)-{R}_{0}\left(1+{{f}}\right){{S}}{{I}}-{{f}}{{S}}$$

14 $$\frac{{{d}}{{I}}}{{{d}}{{\delta}}}={{R}}_{0}\left(1+{{f}}\right){{S}}{{I}}-\left(1+{{f}}\right){{I}}$$

where $\delta =\frac{t}{\gamma }$ is the time and epoch of our model, measured in units of the average infection period, $f=\frac{\mu }{\gamma }$ is part of the average life span and represents the infection period, and ${\mathcal{R}}_{0}=\frac{\beta }{(\gamma +\mu )}$ is the basic infectious individual production number, a measure of the number of individuals in a susceptible group to an infected person who can spread the virus to^51^.

The maximum ${\theta }_{crit}$ value of satisfying these ESS conditions is called the invasion barrier for strategy $V$. If the proportion of strategy $V$ in the population is less than ${\theta }_{crit}$, $P$ cannot penetrate the population. According to these payoffs, the individual’s remuneration to select $P$ at the population level in the early stages of the epidemic can be expressed as Eq. (15)^49^.

15 $${{{\theta}}}_{{{c}}{{r}}{{i}}{{t}}}=\left\{\begin{array}{c}0,\,\,\,\,\,\,\,\,\,\,\,\,\,\,\,\,\,\,\,\,\,\,{\mathcal{R}}_{0}<1\\ 1-\frac{1}{{\mathcal{R}}_{0}},\,\,\,\,\,\,\,\,{\mathcal{R}}_{0}\ge 1\end{array}\right.$$

If $P\ge {\theta }_{crit}$, the epidemiological system converges to the disease-free state $\left(\widehat{S}, \widehat{I}\right)=(1-P, 0)$, whereas if $P<{\theta }_{crit}$, it converges to a stable endemic state, as shown in Eqs. (16) and (17).

16 $$\widehat{{{S}}}=1-{{{\theta}}}_{{{c}}{{r}}{{i}}{{t}}}$$

17 $$\widehat{{{I}}}=\frac{{{f}}}{1+{{f}}}({{{\theta}}}_{{{c}}{{r}}{{i}}{{t}}}-{{P}})$$

Because $S$ and $I$ are constant in this case, the probability of infection of a person who has not been vaccinated can be expressed by using Eq. (18).

18 $${{{\beta}}}_{{{U}}}{{I}}=\frac{{\mathcal{R}}_{0}(1+{{f}})\widehat{{{S}}}\widehat{{{I}}}}{{\mathcal{R}}_{0}\left(1+{{f}}\right)\widehat{{{S}}}\widehat{{{I}}}+{{f}}\widehat{{{S}}}}=1-\frac{1}{{\mathcal{R}}_{0}(1-{{\theta}})}$$

Therefore, the condition $r<{\beta }_{D}I$ for generating a mixed ESS can be written as Eq. (19).

19 $${\mathcal{R}}_{0}\left(1-{{r}}\right)>1$$

The value of mixed ESS ${P}^{*}$ is obtained by solving $r ={\beta }_{U}I{P}^{*}$. In situations where vaccination is perceived as an infection risk ($r>1$), the individual is unlikely to be vaccinated without the help of the model. According to our game theory analysis, considering the condition of ${\mathcal{R}}_{0}\left(1-P\right)>1$ or ESS, the threshold for the infectious disease control vaccination rate at which the individual can stop immunisation is shown in Eq. (20).

20 $${{{P}}}^{\mathbf{*}}=1-\frac{1}{{\mathcal{R}}_{0}\left(1-{{r}}\right)}$$

Based on Eq. (19), the threshold of the perceived relative risk of vaccination to infection when the individual should stop immunising depends on the basic reproduction number ${{R}}_{0}$.

### Normative interpretation of game theory

**Figure 9:**
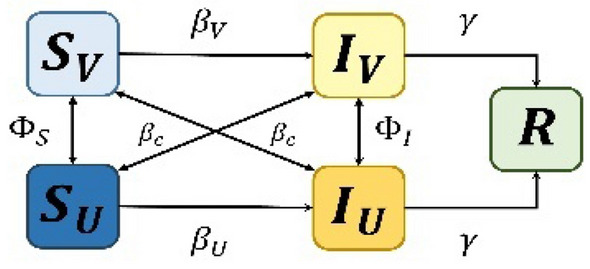
Behavioural epidemiology model for analysing technical intervention of game theory, a model that considers modification of the individual's strategy. ${{V}}$ denotes the vaccinated group, and ${{U}}$ denotes the unvaccinated group. The individual changes the strategy according to the strategy conversion rate ${{\Phi}}$. We classified ${{{\beta}}}_{{{V}}}$ and ${{{\beta}}}_{{{U}}}$ because the infection rate differs as per the strategy. Accordingly, each group was divided according to the strategy; hence, the model was divided into five compartments: ${{{S}}}_{{{V}}},\boldsymbol{ }\boldsymbol{ }{{{S}}}_{{{D}}},\boldsymbol{ }{\boldsymbol{ }{{I}}}_{{{V}}},\boldsymbol{ }\boldsymbol{ }{{{I}}}_{{{U}}},\boldsymbol{ }\boldsymbol{ }{{R}}$*.* The individuals from different strategies may meet. Therefore, we expressed this as the cross-infection rate,${{{\beta}}}_{{{c}}}$

By using the SIR model from a demographic perspective, appropriate vaccine thresholds can be derived. However, this model does not consider the variables for strategy transition^52^. Thus, from a microscopic perspective, we proposed an SIR model that considered the individual's strategy transition, as shown in Fig. 9.

In the proposed model, we considered three infection rates: ${\beta }_{U}$ in the unvaccinated group, ${\beta }_{V}$ in the vaccinated group, and ${\beta }_{c}$ in the contact between these two individual groups. We assumed that the probability ${\beta }_{V}$ of infection of the individual who cooperates with the vaccination is less than the infection rate ${\beta }_{U}$ of the individual (unvaccinated group) trying to obtain the benefits of vaccination without effort (${\beta }_{V}<{\beta }_{U}$). Vaccinated individuals form neutralising antibodies and are less likely to be infected than nonvaccinated individuals. We also defined ${\beta }_{c}$ as the cross-interaction between the two types of strategies ($V\leftrightarrow U$).

We proposed a model incorporating evolutionary game theory and dynamics models by using a parcel approach. The probability that an individual who adopts $i$ according to the general evolutionary game dynamics changes strategy to $j$ is related to its respective remunerations (${\pi }_{i}$ and ${\pi }_{j}$). The probability can be expressed using the Fermi rule as Eq. (21) ^53^:

21 $${{\Theta}}\left({{{\pi}}}_{{{i}}},\boldsymbol{ }{{{\pi}}}_{{{j}}}\right)=\frac{1}{1+{{{e}}}^{-({{{\pi}}}_{{{j}}}-{{{\pi}}}_{{{i}}})/{{k}}}}$$

This is the probability of enabling strategy modification, and the irrationality of changing these strategies is measured by using the parameter $k$. In this study, $k$ = 0.5 was adopted as a constant. Through this, it was possible to determine the ratio of individuals who modified strategies from $i$ to $j$, that is, the strategy conversion rate (Eqs. (22) and (23)).

22 $${{\Phi}}_{{{S}}}={{{S}}}_{{{V}}}\left({{{S}}}_{{{U}}}+{{{I}}}_{{{U}}}\right){\Theta }\left({{{\pi}}}_{{{V}}},{{{\pi}}}_{{{U}}}\right)-{{{S}}}_{{{U}}}\left({{{S}}}_{{{V}}}+{{{I}}}_{{{V}}}\right){\Theta }({{{\pi}}}_{{{U}}},{{{\pi}}}_{{{V}}})$$

23 $${{\Phi }}_{{{I}}}={{{I}}}_{{{V}}}\left({{{S}}}_{{{U}}}+{{{I}}}_{{{U}}}\right){\Theta }\left({{{\pi}}}_{{{V}}},{{{\pi}}}_{{{U}}}\right)-{{{I}}}_{{{U}}}\left({{{S}}}_{{V}}+{{{I}}}_{{{V}}}\right){\Theta }\left({{{\pi}}}_{{{U}}},{{{\pi}}}_{{{V}}}\right)$$

In relation to infection dynamics, an infection rate of ${\beta }_{V}<{\beta }_{c}<{\beta }_{U}$ exists in general situations. Here, ${\beta }_{c}$ is defined as ${\beta }_{c}=c({\beta }_{V}+{\beta }_{U})/2$. The crossing parameter $\mathrm{c}$ between the $V$ and $U$ is within the range of $0<c<1$, and in this study, $c=0.1$ was assumed because of the high possibility of cross-infection owing to vaccine pass release. The differential equations of the SIR vacuum model that consider all the following assumptions are Eqs. (24, 25, 26, 27, 28)^52^.

24 $$\dot{{{{S}}}_{{{U}}}}=-{{{S}}}_{{{U}}}\left({{{\beta}}}_{{{U}}}{{{I}}}_{{{U}}}+{{{\beta}}}_{{{c}}}{{{I}}}_{{{V}}}\right)+{{{\zeta}}{{\Phi}}}_{{{S}}}$$

25 $$\dot{{{{S}}}_{{{V}}}}=-{{{S}}}_{{{V}}}\left({{{\beta}}}_{{{c}}}{{{I}}}_{{{U}}}+{{{\beta}}}_{{{V}}}{{{I}}}_{{{V}}}\right)-{{{\zeta}}{{\Phi}}}_{{{S}}}$$

26 $$\dot{{{{I}}}_{{{U}}}}={{{S}}}_{{{U}}}\left({{{\beta}}}_{{{U}}}{{{I}}}_{{{U}}}+{{{\beta}}}_{{{c}}}{{{I}}}_{{{V}}}\right)-{{\gamma}}{{{I}}}_{{{U}}}+{{{\zeta}}{{\Phi}}}_{{{I}}}$$

27 $$\dot{{{{I}}}_{{{V}}}}={{{S}}}_{{{V}}}\left({{{\beta}}}_{{{c}}}{{{I}}}_{{{U}}}+{{{\beta}}}_{{{V}}}{{{I}}}_{{{V}}}\right)-{{\gamma}}{{{I}}}_{{{V}}}-{{{\zeta}}{{\Phi}}}_{{{I}}}$$

28 $$\dot{{{R}}}={{\gamma}}({{{I}}}_{{{U}}}-{{{I}}}_{{{V}}})$$

In the normative interpretation of game theory, dynamic scenarios based on behaviour can be analysed by using the game-theory-based SIR dynamics model. In this study, parameters ${r}_{V}=1, {r}_{i}=10, \zeta =1, \gamma =1.25, {\beta }_{V}=1, {\beta }_{U}=10, k=0.5,c=0.1$ were adopted as constant variables, and the variations in the infection risk ${r}_{i}$ and infection rate ${\beta }_{U}$ of the unvaccinated group were analysed^52^. In this case, ${I}_{0}=0.01$ and ${S}_{0}=1-{I}_{0}$ were set between the $V$ and $U$ strategies because the number of infected individuals was minimal at the beginning of the infection. Simultaneously, we described an ideal situation in which a person adopting the V strategy was vaccinated with the probability of ${P}^{*}$.

Each individual can receive an optimal reward if the least selected strategy was selected on average. This payoff matrix can be considered a noncooperative game, and the best strategy in a noncooperative situation is to reverse what the other party does^54^. This is like the problems presented in vaccination. Individuals should be vaccinated; however, if a majority of the population is vaccinated, an individual’s motivation to not get vaccinated increases. The noncooperative factor is related to the risk of a relative vaccine against infection risks. Moreover, the risk of infection ${r}_{i}$ fluctuates based on the infected individual and is a central medium for attaining a continuous infection peak.

**Figure 10:**
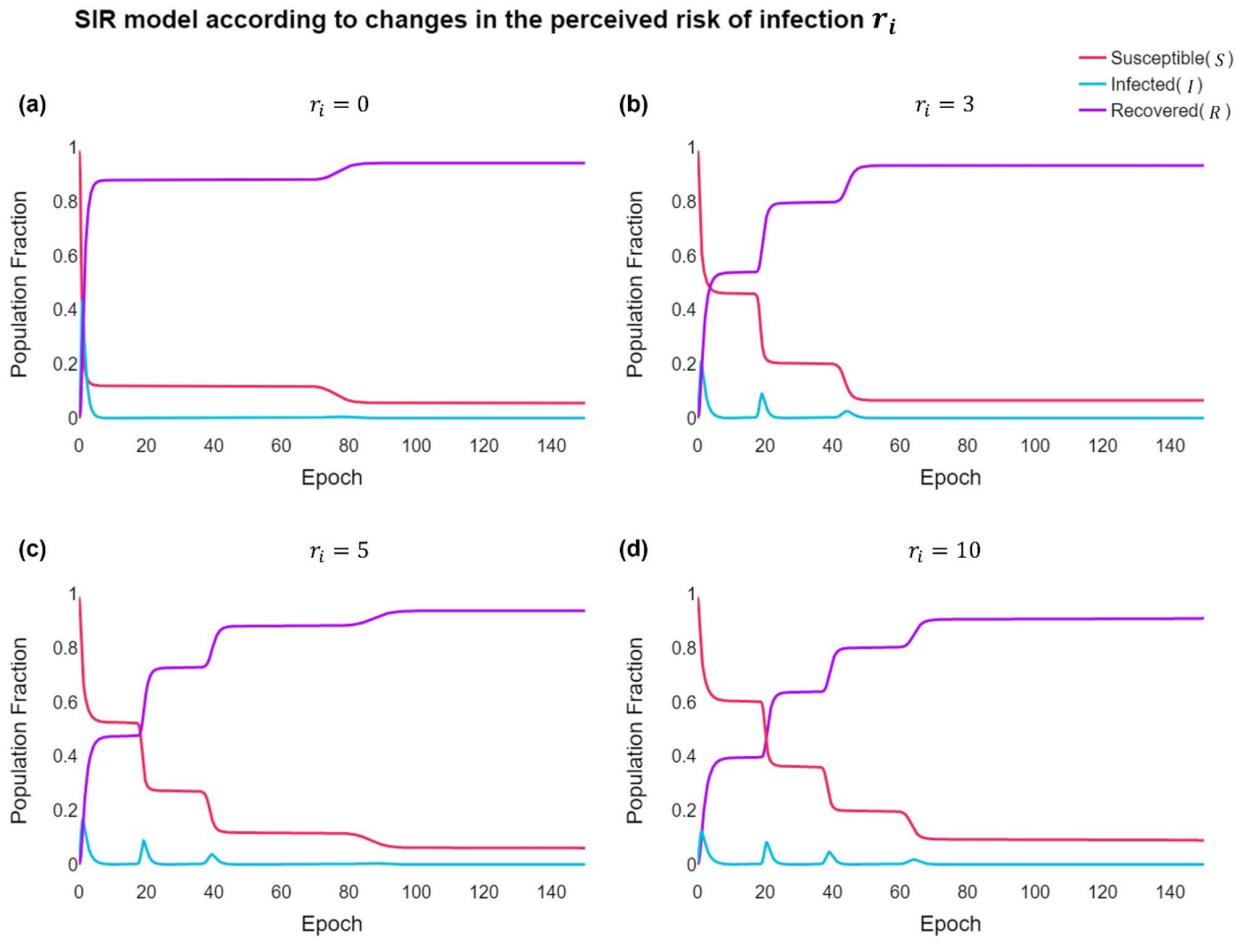
SIR model according to changes in the perceived risk of infection (**A**) ${r}_{i}=0$, (**B**) ${r}_{i}=3$, (**C**) ${r}_{i}=5$, and (**D**) ${r}_{i}=10$. As the general behavioural dynamics of the epidemic population ${{S}}=({{{S}}}_{{{V}}}+{{{S}}}_{{{U}}})$, ${{I}}=({{{I}}}_{{{V}}}+{{{I}}}_{{{U}}})$, ${{R}}$, and ${{{r}}}_{{{i}}}$ increase, the individual’s strategy frequently changes. Based on variations in the infection wave for various disease recognition values ${{{r}}}_{{{i}}}$, the initial infection peak is low if the value is high, and a vibration term is generated to disperse the number of confirmed patients. This parameter significantly influences the infection peak size, decreasing it while spreading the cases across several smaller infection waves.

When the risk of infection is low, the individual feels that the risk of the vaccine is greater than that of the infection. Thus, the individual withdraws the $V$ strategy and adopts the $U$ strategy within a short period, as shown in Fig. 10. In this case, many people become infected within the same time, leading to shortages of beds and medical personnel and more damage. However, when the risk of infection is high, the perception of risk of infection increases as the peak of infection persists. Consequently, the payoff for vaccination and the number of individuals cooperating with it increase. The proportion of simultaneous infections decreases, increasing the possibility of preventing a pandemic. In other words, it is possible to have time to suggest pre-emptive measures to adjust the peak size of simultaneously infected people when infectious diseases spread. Therefore, to prevent simultaneous infections through vaccination, we should not only emphasise the importance of vaccines but also the altruistic attitude to form herd immunity, giving individuals a reason to get vaccinated to increase the payoff of relative vaccines.

Additionally, ${r}_{i}$ influences the variance in confirmed cases. When ${r}_{i}$ is low, the number of infected people increases exponentially over a short period. However, as ${r}_{i}$ increases, the distribution of confirmed cases is dispersed, and multiple oscillation terms exist. However, there may be insufficient confirmed cases to paralyze the medical system. We changed the following values to clarify the infection risk perception for the size and duration of infection peaks, which greatly rely on ${r}_{i}$.

Therefore, the size of the infection peak is critical when investigating the epidemiology of an epidemic. The analysis results for the variations in ${I}_{max}$ with ${r}_{i}$ and ${\beta }_{U}$ are shown below. That is, the size of ${I}_{max}$ increases as ${\beta }_{U}$ increases but decreases as ${r}_{i}$ increases. Therefore, the higher the risk of infection is, the higher the individual's benefit from the vaccine, indicating that the individual exhibits a more cooperative attitude towards the vaccine; hence, herd immunity can be rapidly achieved.

The probability ${\beta }_{U}I$ that an individual who chooses the $U$ strategy is infected should decrease as $\theta$ increases until $\theta$ reaches ${\theta }_{crit}$. Currently, all parameters are greater than 0. Thus, the following maximum expected reward values are obtained by using Eq. 4 when $P=1$ (always vacuum), the maximum expected payoff can be obtained if ${\beta }_{U}I>r$, and the maximum expected payoff can be obtained if ${\beta }_{U}I<r$ when $P=0$ (always unvacuumed). Therefore, we can define the strategy change point $I^{\prime}$ as Eq. (29).

29 $${{{I}}}^{{^{\prime}}}=\frac{{{{r}}}_{{{v}}}}{{{{r}}}_{{{i}}}{{{\beta}}}_{{{U}}}}$$

The vaccinated group ratio can be expressed as $V=({S}_{V}+{I}_{V})/(S+I)$. Since only two strategies exist in the dynamics model, it can be expressed as $U=1-V$. The rate of change of strategy varies with strategy flux terms ${\Phi }_{S}$ and ${\Phi }_{I}$; that is, the rate of change of strategy *V* ˙ can be expressed as Eq. (30).

30 $$\dot{{{V}}}={ }{-{{\Phi}}}_{{{S}}}-{{{\Phi}}}_{{{I}}}=\left({{{S}}}_{{{U}}}+{{{I}}}_{{{U}}}\right)\left({{{S}}}_{{{V}}}+{{{I}}}_{{{V}}}\right){{\Theta}}\left({{{\pi}}}_{{{U}}},{{{\pi}}}_{{{V}}}\right)-{ }\left({{{S}}}_{{{V}}}+{{{I}}}_{{{V}}}\right)\left({{{S}}}_{{{U}}}+{{{I}}}_{{{U}}}\right){{\Theta}}({{{\pi}}}_{{{V}}},{{{\pi}}}_{{{U}}})$$

If we rearrange $\dot{V}$ according to $V(S+I)=({S}_{V}+{I}_{V})$, $U(S+I)=({S}_{U}+{I}_{U})$, and $S+I+R=1$, the expression is Eq. (31).

31 $$\dot{{{V}}}={\left(1-{{R}}\right)}^{2}{{V}}{{U}}[{{\Theta}}\left({{{\pi}}}_{{{U}}},{{{\pi}}}_{{{V}}}\right)-{{\Theta}}\left({{{\pi}}}_{{{V}}},{{{\pi}}}_{{{U}}}\right)]$$

Here, ${\left(1-R\right)}^{2}$ controls the rate of change of the strategy because it relates to the total available population, which can vary its strategy. However, the most critical factor is the remainder of the equation. This is the general mean-field form of the master equation for the evolution of cooperation in two-strategy games. The proposed model is consistent with and returns to an evolutionary game considering only the strategy density.

## Supplementary Information

Supplementary Figures.
