# Supplementary material for: Attitudinal analysis of vaccination effects to lead endemic phases
Source: Sci Rep. 2023 Jun 24;13:10261. doi: 10.1038/s41598-023-37498-y (PMC10290696; doi:10.1038/s41598-023-37498-y)
Supplement: Supplementary file 1 — Supplementary Figures. [file 41598_2023_37498_MOESM1_ESM.docx]

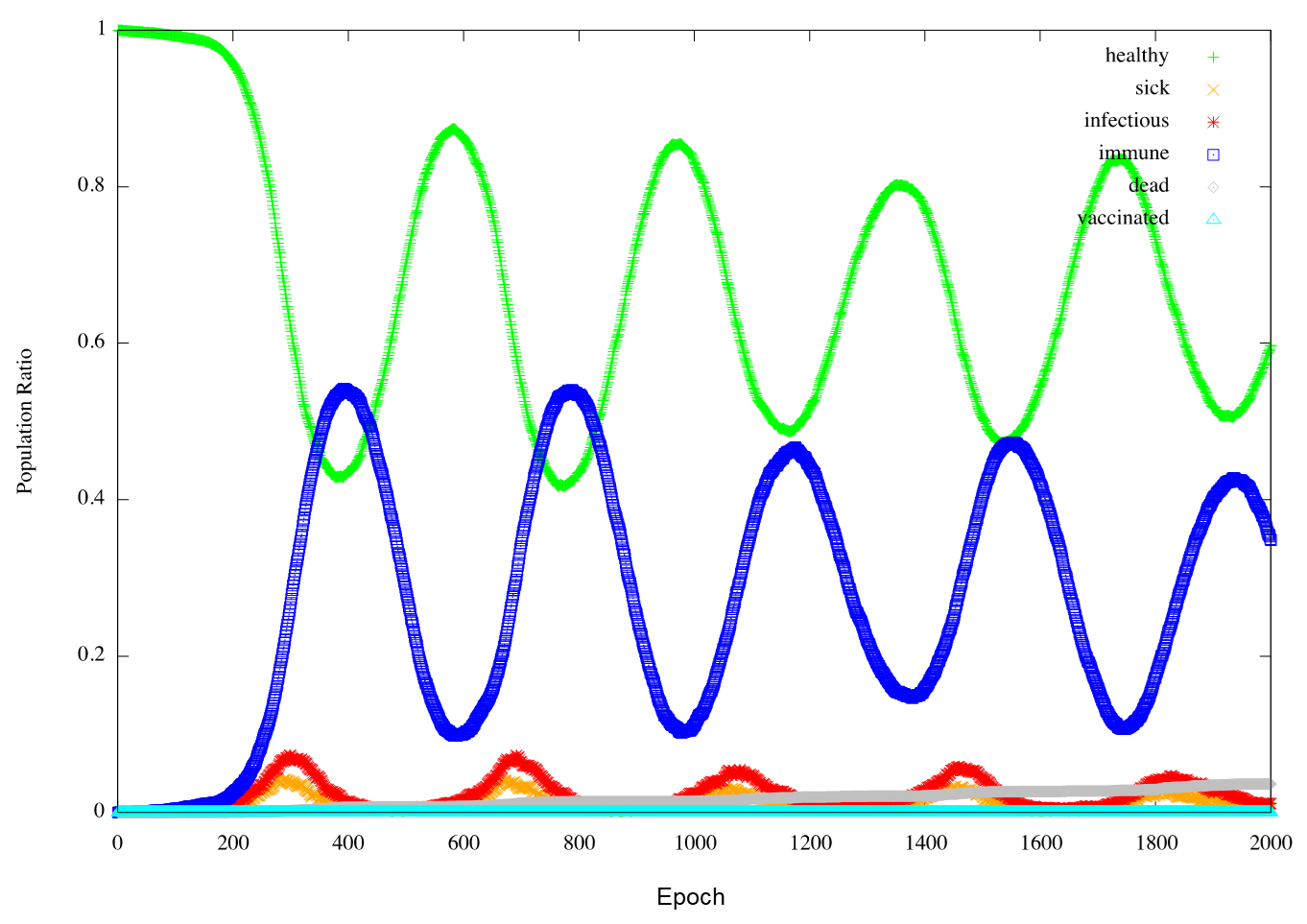


Supplementary Figure 1. the population ratios of various types of individuals when $\boldsymbol{\rho}\boldsymbol{=0.08}$ $\boldsymbol{m}^{\boldsymbol{-2}}$ and $\boldsymbol{p}_{\boldsymbol{vacc}}\boldsymbol{=0}$. Even though the parameters used in the IBMC simulations look somewhat arbitrary, the presence of the recurrent pattern observed in the simulations is rather insensitive to the parameter space. The animation of the IBMC simulation when $\boldsymbol{p}_{\boldsymbol{vacc}}\mathbf{=0.4}$ : Green, organ, red, blue, grey, and cyan colours correspond to healthy, sick but not infectious, infectious, immune, dead, and vaccinated individuals, respectively.
